# Harnessing virtual reality in pain management: a call for standardization and clinical guidelines

**DOI:** 10.1097/MS9.0000000000004065

**Published:** 2025-10-09

**Authors:** Chukwuka Elendu, Daniel E. Otobrise, Courage O. Idahor, Victor I. Orji, Chima P. Igbokwe

**Affiliations:** a Division of Medicine, Federal University Teaching Hospital, Owerri, Nigeria; b Division of Medicine, University of Medical Sciences, Ondo, Nigeria; c Division of Medicine, Henry Ford Health, Detroit, Wayne County, Michigan, USA; d Division of Medicine, Lagos University Teaching Hospital, Idi-Araba, Nigeria; e Division of Medicine, Abia State University, Uturu, Nigeria

*Dear Editor,*

We read with great interest the recently published article by Masood *et al, Virtual reality as a transformative tool in pain management*^[1]^. The authors comprehensively highlight the evolving role of virtual reality (VR) in both acute and chronic pain contexts, underscoring its potential to reshape analgesic strategies. As VR continues to gain attention as a scalable non-pharmacological tool, it is essential to emphasize not only its therapeutic promise but also the challenges that must be addressed to ensure its safe, equitable, and evidence-based integration into clinical practice.

Pain remains a major clinical and public health burden, with far-reaching effects on quality of life and healthcare utilization^[2]^. Conventional pharmacological approaches, particularly opioids, are limited by risks of dependency, tolerance, and adverse events; searching for safe adjuncts is an urgent priority^[3]^. The evidence presented in the article supports the analgesic potential of VR, which operates by simultaneously modulating sensory, cognitive, and emotional dimensions of pain perception. Functional neuroimaging studies demonstrate that VR activates brain regions involved in cognitive regulation and emotional modulation, while reducing activity in nociceptive processing pathways^[4]^. This multidimensional action profile sets VR apart from many other non-pharmacological interventions, as summarized in Table 1, which highlights current evidence, mechanisms, barriers, and future directions.

**Table 1:** Summary of current evidence, mechanisms, barriers, and future directions for virtual reality in pain management

Clinical context	Key findings	Reported pain reduction	Mechanistic basis	Advantages over standard care	Barriers/limitations	Research gaps	Future directions
Burn wound care	VR distraction during procedures	35–50% reduction^[5]^	Modulation of sensory and emotional pain pathways	Superior to standard distraction	Small sample sizes; device cost	Larger RCTs; standardized VR content	Integration into wound care protocols
Pediatrics	Procedural pain (e.g., venipuncture)	Reduced self-reported pain and distress^[2,5]^	Immersive presence reduces anxiety	May reduce sedation/anesthesia needs	Heterogeneity in interventions	Long-term safety/effectiveness data	Pediatric-specific VR guidelines
Chronic pain (fibromyalgia, CRPS, neuropathic pain)	Sustained improvement in intensity and function^[2,3]^	Variable (moderate reduction)	Restores cortical representation; reduces central sensitization	Addresses maladaptive neuroplasticity	Limited follow-up duration	Comparative studies vs. standard therapies	Incorporation into multimodal programs
Obstetric populations	Labor pain management	Decreases in sensory, affective, and cognitive domains^[4]^	Distraction + emotional regulation	Reduces anxiety during labor	Limited trial numbers	Standardized trial designs	Integration into maternity care
Mechanistic studies	Motor imagery and feedback and graded exposure	Not quantified	Targets cortical reorganization and anxiety avoidance	Goes beyond analgesia	Translational gap between lab and clinic	Mechanistic validation in diverse groups	AI-driven adaptive VR therapies
Health system impact	Potential opioid-sparing effect	Indirectly reduced	Reduced opioid reliance and admissions	Cost savings potential	High upfront technology cost	Lack of cost-effectiveness data	Economic modeling and health policy integration
Implementation challenges	Practical barriers	N/A	N/A	Equity concerns in tech access	Cost, training, literacy, and infrastructure	Few guidelines on implementation	Clinician training and patient education programs
Research priorities	Multicenter, standardized RCTs	N/A	Comparative validation	Establishes credibility	Current evidence fragmented	Lack of standardized protocols	Guideline development and global consensus

VR, virtual reality; RCT, randomized controlled trial; CRPS, complex regional pain syndrome.

Clinical studies across diverse populations provide encouraging results, with VR interventions during burn wound care demonstrating a 35–50% reduction in reported pain intensity and outperforming standard distraction methods^[5]^. In pediatric settings, VR has shown efficacy in procedural pain, reducing both self-reported pain and behavioral distress during venipuncture and other interventions^[2,5]^. These findings are particularly relevant in minimizing the need for general anesthesia or sedation in children undergoing minor procedures^[1]^. In chronic pain conditions, repeated VR sessions have yielded sustained improvements in pain intensity and function in fibromyalgia, complex regional pain syndrome (CRPS), and neuropathic pain^[2,3]^. Furthermore, trials in obstetric populations suggest VR reduces labor-related sensory, affective, and cognitive pain domains, as well as anxiety^[4]^. Such broad applicability makes VR a versatile adjunct across the pain continuum.

Mechanistically, VR offers unique therapeutic advantages by creating an immersive sense of presence that facilitates graded exposure for pain-related anxiety and avoidance, thereby supporting psychological adaptation^[2]^. Motor imagery and real-time visual feedback delivered via VR platforms can help restore altered cortical representations, which is particularly valuable in phantom limb pain and CRPS^[3]^. These mechanisms extend beyond analgesia alone, targeting the maladaptive processes of central sensitization and body perception disturbance that often perpetuate chronic pain.

Despite these benefits, key barriers must be addressed before VR can be integrated into routine pain management. First, many existing studies, though promising, are limited by small sample sizes, heterogeneity of patient populations, and variability in intervention protocols^[4]^. Without large, well-designed randomized controlled trials (RCTs), it does not remain easy to draw firm conclusions about comparative effectiveness against conventional modalities. Second, there is a lack of standardized treatment parameters, including optimal session length, frequency, and type of VR content, which hinders reproducibility and generalizability across clinical settings. Third, practical challenges such as the cost of head-mounted displays, the need for technical literacy, and limited infrastructure in resource-constrained environments restrict equitable access. Although technology costs are decreasing, the digital divide in healthcare delivery remains a real concern^[5]^. These limitations emphasize the importance of developing a structured framework for VR implementation, as outlined in Table 2. Addressing these challenges holds substantial implications, as VR could reduce opioid prescriptions, shorten hospital stays, and lower emergency department utilization for pain crises, thereby easing the burden on healthcare systems^[6]^. Moreover, advances in artificial intelligence and biofeedback may soon enable personalized VR therapies that adapt dynamically to individual patient pain profiles, making VR not just a distraction tool but a precision analgesic modality.

**Table 2:** Proposed framework for standardizing virtual reality in pain management

Domain	Current gaps	Standardization priorities	Practical implementation steps
Clinical indications	Fragmented evidence across acute, chronic, pediatric, and obstetric populations	Define clear evidence-based indications for VR use	Develop consensus guidelines stratified by pain type and setting
Treatment parameters	No standard session length, frequency, or content type	Establish core treatment protocols (duration, frequency, and VR environment design)	Pilot multicenter harmonized RCTs to test feasibility
Comparative effectiveness	Few head-to-head trials vs. standard therapies	Require trials with active comparators (pharmacological, behavioral, and device based)	Incorporate VR arms into existing multimodal pain trials
Cost and access	Technology cost and infrastructure disparities	Cost-effectiveness modeling and reimbursement frameworks	Public–private partnerships to subsidize devices
Training and literacy	Limited clinician familiarity and patient usability concerns	Create structured training modules for healthcare providers	Integrate VR literacy into pain clinics and nursing curricula
Safety and ethics	Sparse long-term safety data and regulatory ambiguity	Define safety endpoints, contraindications, and ethical safeguards	Collaborate with regulatory bodies for VR certification standards
Research methodology	Heterogeneous study designs, small samples, and variable outcomes	Standardize trial design, outcomes, and reporting	Develop global registry for VR pain studies
Future integration	VR used mainly as distraction rather than personalized therapy	Personalization via AI, biofeedback, and adaptive algorithms	Incorporate digital biomarkers for precision VR analgesia

AI, artificial intelligence; VR, virtual reality.

To ensure these benefits are realized, the field urgently requires multicenter RCT with standardized protocols that compare VR directly to existing standard-of-care therapies. Development of clinical guidelines for VR implementation in pain management should follow a similar approach to the frameworks established for other non-pharmacological modalities^[3]^. Furthermore, cost-effectiveness analyses must be incorporated into study designs to evaluate scalability and feasibility for widespread adoption. Finally, training programs for clinicians and patient education strategies are needed to reduce the barriers posed by technology literacy and usability. In this context, Table 3 provides practical recommendations for integrating VR into pain management programs, focusing on patient selection, protocols, training, accessibility, safety, and evaluation.

**Table 3:** Practical recommendations for integrating virtual reality into pain management programs

Implementation area	Key recommendation	Expected benefit	Example application
Patient selection	Identify suitable candidates (e.g., procedural pain, pediatric, and fibromyalgia)	Maximizes therapeutic response	Screening in pain clinics
Clinical protocols	Standardize session length, frequency, and VR environment	Enhances reproducibility	20–30 min sessions and 2–3 times weekly
Training	Provide structured training for clinicians and staff	Improves adoption and safety	Continuing Medical Education (CME) modules for anesthesiologists, nurses
Accessibility	Ensure affordable devices and technical support	Reduces digital divide	Subsidized VR headsets for low-resource settings
Monitoring and safety	Define safety endpoints, side effect monitoring	Promotes patient safety	Tracking cybersickness and dizziness
Evaluation	Include VR outcomes in pain registries	Strengthens evidence base	National pain registries adding VR modules

VR, virtual reality.

The article by Masood *et al*^[1]^ is an essential contribution to the literature, emphasizing the transformative potential of VR in reshaping the pain management paradigm. However, as with many emerging technologies, the risk of premature enthusiasm must be balanced with methodological rigor and systematic implementation. By prioritizing evidence-based protocols, standardization, and accessibility, VR can evolve from a promising adjunct into a validated cornerstone of multimodal pain management strategies.

In conclusion, VR holds significant promise for reducing pain intensity, minimizing opioid reliance, and enhancing patient experience across a range of clinical settings. Yet, the widespread adoption of VR in pain management will depend on our collective ability to establish robust evidence, standardized protocols, and practical guidelines that ensure accessibility across diverse patient populations. Finally, in line with best practices for scientific integrity and the responsible integration of digital tools in research, we acknowledge the TITAN Guidelines 2025 for transparency in the reporting of artificial intelligence while noting that no generative AI tools were used in the preparation of our work^[7]^. We commend the authors for their timely exploration of this topic and encourage further discussion, research, and policy development to harness VR’s full potential in pain management.

## Data Availability

All data referenced in this letter are from publicly available sources cited in the article. No original data were collected.
